# Epidemiological characteristics of hearing loss associated with noise temporal structure among manufacturing workers

**DOI:** 10.3389/fnint.2022.978213

**Published:** 2022-09-08

**Authors:** Lifang Zhou, Xiaoying Ruan, Tongshuai Wang, Hongwei Xie, Yong Hu, Zhihao Shi, Jiarui Xin, Jiena Zhou, Panqi Xue, Fang Wei, Yixin Zhang, Meibian Zhang, Hua Zou

**Affiliations:** ^1^Institute of Occupational Health and Radiation Protection, Zhejiang Provincial Center for Disease Control and Prevention, Hangzhou, China; ^2^Department of Occupational Health, Hangzhou Hospital for Prevention and Treatment of Occupational Disease, Hangzhou, China; ^3^Clinical and Translational Research Center, Tongji University School of Medicine, Shanghai, China; ^4^Jiaxing Center for Disease Control and Prevention, Jiaxing, China; ^5^National Institute of Occupational Health and Poison Control, Beijing, China; ^6^Department of Public Health, Zhejiang University School of Medicine, Hangzhou, China; ^7^School of Medicine, Hangzhou Normal University, Hangzhou, China

**Keywords:** noise, complex noise, hearing loss, manufacturing industry, epidemiological characteristics

## Abstract

**Objective:** This study aimed to investigate the epidemiological characteristics of occupational noise-induced hearing loss (NIHL) among manufacturing workers, and to provide evidence for diagnosing and preventing occupational hearing loss caused by complex noise, which is different from Gaussian noise in temporal structure.

**Methods:** One thousand and fifty manufacturing workers exposed to occupational noise were recruited in a cross-sectional survey. Exposure characteristics and epidemiological distribution of hearing loss and noise exposure metrics (noise energy and kurtosis) were investigated, and the relationship between noise exposure and hearing loss was analyzed. The effects of kurtosis on hearing threshold shift across different frequencies and on NIHL development with exposure duration and noise intensity were also investigated.

**Results:** Each type of work had specific noise exposure metrics. Noise intensity and kurtosis were independent parameters (*r* = −0.004, *p* = 0.885). The prevalence of NIHL and the hearing threshold level had a specific distribution in different types of work. Kurtosis deepened the hearing notch at high frequencies and accelerated the formation of early hearing loss. The effect of exposure duration and noise intensity on the prevalence of high-frequency NIHL (i.e., at 3, 4, 6, and 8 kHz) for manufacturing workers increased with kurtosis in workers with noise exposure duration of less than 10 years and with L_Aeq.8h_ between 80 and 90 dB(A). Male (OR = 1.557, 95%CI = 1.141–2.124), age (OR = 1.033, 95%CI = 1.014–1.052), exposure duration (OR = 1.072, 95%CI = 1.038–1.107), kurtosis (OR = 1.002, 95%CI = 1.001–1.003), and noise intensity (L_Aeq.8h_; OR = 1.064, 95%CI = 1.044–1.084) were risk factors for high-frequency NIHL. The speech-frequency NIHL (i.e., at 0.5, 1, and 2 kHz) risk of workers exposed to manufacturing noise was related to age (OR = 1.071, 95%CI = 1.043–1.100). There were no statistically significant associations between speech-frequency NIHL and sex, noise exposure duration, kurtosis, and noise intensity (L_Aeq.8h_).

**Conclusion:** The high-frequency NIHL prevalence among manufacturing workers is associated with sex, age, exposure duration, noise intensity, and temporal structure of noise, while the speech-frequency NIHL prevalence is associated with age. Kurtosis strengthens the association of noise exposure duration and noise intensity with high-frequency hearing loss. The influence of noise temporal structure should be considered in the diagnosis and early prevention of occupational hearing loss caused by complex noise.

## Introduction

Over 5% of the world’s population (i. e., 430 million people) suffer from deafness and hearing loss (WHO, 2021). Exposure to occupational noise is one of the most common risks for hearing loss in China and across the world. About 16% of adult hearing loss cases are associated with occupational noise exposure (Nelson et al., 2005). Noise-induced hearing loss (NIHL) is the second leading cause of sensorineural hearing loss (Chen et al., 2019). Occupational NIHL is the most prevalent occupational disease among working-age people worldwide (Chen et al., 2020). In China, occupational NIHL ranks as the second primary occupational disease with an annual increase of approximately 20% (Zhou et al., 2020).

In terms of its temporal structure, industrial noise can be divided into Gaussian noise (i.e., steady-state, continuous noise) and non-Gaussian noise, also known as complex noise (Qiu et al., 2006). Complex noise consists of transient high-energy impulsive noise superimposed on Gaussian background noise. The characteristics of hearing loss based on noise energy are well understood. With the development of industrialization, non-Gaussian noise has been the most prevalent type of noise in working environments (Hamernik and Qiu, 2001). Researchers proposed that the equal energy hypothesis (EEH; ISO, 2013) in the existing international noise exposure standards (e.g., ISO 1999:2013) might not be adequate for complex noise evaluation (Zhang et al., 2021a). One of the risk factors that may contribute to the high incidence of occupational NIHL is considered to be the damage-risk criteria for noise exposure relying only on energy-based exposure assessment (Davis et al., 2009).

In recent years, hearing loss caused by complex noise has become a hotspot worldwide. An energy metric alone should not be adequate to predict the risk of NIHL (Davis et al., 2009). The previous animal experiments and epidemiological studies demonstrated that the temporal structure of noise was an additional metric to assess the hearing loss caused by complex noise (Qiu et al., 2006; Davis et al., 2012; Suter, 2017). Studies showed that the noise temporal structure is a risk factor for NIHL, and complex noise has a much greater impact on hearing loss than Gaussian noise (Dunn et al., 1991; Hamernik and Qiu, 2001; Zhou et al., 2020; Shi et al., 2021). Recently, evidence has shown that the temporal structure of complex noise can be expressed in the kurtosis metric (β), which is defined as the ratio of the fourth-order central moment to the squared second-order central moment of a distribution (Davis et al., 2009; Zhao et al., 2010; Davis and Clavier, 2017). For a fixed range of noise exposure level and duration, the noise-induced permanent threshold shifts (NIPTS) increased with the kurtosis of the noise (Zhang et al., 2021c).

Studies have indicated that the prevalence of NIHL increased with exposure duration, noise energy levels, sex, and age (Lie et al., 2014; Chen et al., 2019; Zhou et al., 2020; Zhang et al., 2021b). A total of 24.4% of adults had an audiometric notch in the United States, this was more common among males than females (Carroll et al., 2017). The prevalence of NIHL was higher in workers who experienced prolonged exposure and older workers in textile industries (Abraham et al., 2019). However, the epidemiological characteristics of occupational NIHL related to the kurtosis metric of complex noise have been much less explored. In this study, the noise exposure and hearing loss of workers in the textile, furniture, and general equipment manufacturing industries were investigated. We analyzed the epidemiological characteristics (especially those associated with the temporal structure of noise) of occupational hearing loss caused by complex noise in the manufacturing industry to help provide a basis for diagnosis and early prevention of occupational hearing loss caused by complex noise.

## Materials and Methods

### Subjects

From 2017 to 2019, we carried out a cross-sectional survey of the manufacturing industry in Zhejiang province, China. The cluster sampling method was used to recruit noise-exposed workers from four textile enterprises, six furniture manufacturers, and eight general equipment manufacturing enterprises. Each participant was asked to sign an informed consent form after being informed of the purpose of this study. The participants met the following requirements, which were determined from the noise exposure questionnaire: (1) working in the same type of work in the current factory; (2) no history of another high-level noise exposure except for the current job, including occupational and non-occupational noise exposure; (3) no co-exposure history of noise and ototoxic organic solvents or heavy metals; (4) self-reported never using ototoxicity drug; (5) never suffered from ear diseases; (6) no diabetes; (7) never had military service or shooting experience; (8) no or minimal use of hearing protection devices (HPD). The Medical Ethics Committee of Zhejiang Center for Disease Control and Prevention approved the study protocol (approval reference number: ZJCDC-T-043-R), which met the ethical requirements.

Finally, we enrolled 1,050 workers who met the study’s inclusion criteria. The workers were divided into different groups by types of work, which included spinners, weavers, roller operators in the textile industry, gun nailers and carpenters in the furniture manufacturing industry, and assemblers, metal processing workers, welders, polishers, forgers, stampers, and carvers in the general equipment manufacturing industry.

### Field investigation and questionnaire survey

A field investigation in workplaces was conducted to get information on devices, materials, products, production processes, the number of workers exposed to the noise, the distribution of noise sources, and measures taken to reduce the noise level of each factory. The questionnaire designed by the research team was used to conduct the face-to-face questionnaire survey of all participants by occupational hygienists. There were eight occupational hygienists in our research team, who were responsible for conducting the questionnaire, and they were trained to standardize their understanding of the questionnaire. Each worker was assisted by an occupational hygienist to complete the questionnaire. The questionnaire collected the following information from the participants: (1) general personal information and lifestyle (e.g., age, sex, smoking, and alcohol use); (2) health conditions and medical history: blood pressure, complaints of hearing impairment, history of ear diseases and hearing loss, history of other diseases (chronic diseases, traumatic brain injury, mumps, scarlet fever, measles, etc.), surgical history, and use of ototoxic drugs (gentamicin, streptomycin, clarithromycin, quinine, etc.); (3) occupational history, such as industry, factory, workshop, type of work, noise exposure duration (ED), chemical exposure at work, and HPD use, including information of current and previous work; (4) non-occupational noise exposure (e.g., frequency and duration of recreational noise exposure); (5) other information (military service or shooting behavior, family history of hearing loss, etc.).

### Noise exposure measurement

The digital individual noise recorder (ASV5910-R, Hangzhou Aihua Instruments Co., Ltd., China) that can measure noise from 40 dB(A) to 141 dB(A) was used to record a shift-long personal noise exposure for each participant. The recorder uses a pre-polarized condenser microphone with a broad response frequency (20 Hz to 20 kHz) and high sensitivity level (2.24 mV/Pa). The microphone was placed on the shoulder of each participant during the whole work shift.

The A-weighted noise exposure level normalized to a nominal 8-h working day (L_Aeq.8h_) and kurtosis of noise (β) were used to quantify noise exposure in this study. The MATLAB software was used to analyze the shift-long noise and obtain the L_Aeq.8h_ and kurtosis. The L_Aeq.8h_ level was calculated by the formula in ISO 1999 (ISO, 2013):


(1) $$L_{Aeq,8h}=L_{Aeq,Te}+10\times \mathrm{lg}\left(\frac{T_{e}}{T_{0}}\right)$$

Where T_e_ is the effective duration of the working day in hours; T_0_ is the reference duration (T_0_ = 8 h); and L_Aeq,Te_ is the L_Aeq_ for T_e_. The kurtosis values were computed over consecutive 40-s time windows without overlap over the shift-long noise record using a sampling rate of 48 kHz. The mean of the kurtosis values was then calculated to be the kurtosis metric in this study.

The occupational exposure limit (OEL) of workplace noise level is 85 dB(A) in China. Then we divided noise levels into four groups according to L_Aeq.8h_: <80, 80–85, 85–90, and ≥ 90 dB(A). This study set *β* = 10 as a boundary to distinguish complex noise from steady-state noise (Davis et al., 2009). Furthermore, we divided complex noise into two groups of 50.

### Hearing loss determination

#### Audiometric test

Pure tone air conduction hearing threshold measurements at the speech frequencies (i.e., 0.5, 1, and 2 kHz) and the high frequencies (i.e., 3, 4, 6, and 8 kHz) at both ears were performed after excluding conductive hearing impairments by general ear examination of each participant. The participants were out of the occupational noise environment for at least 16 h before the test. The audiometric test was performed in an audiometric room of a mobile physical examination vehicle using an audiometer (Interacoustics AD629, Denmark) with an air conduction headphone (HDA300), which was calibrated by the Zhejiang Institute of Metrology according to the Chinese standard (Verification Regulation of Audiological Equipment Pure-tone Audiometers, JJG 388-2012). The NIPTS at each frequency for each participant were obtained according to Annex A of ISO 1999 (ISO, 2013). Measured hearing threshold levels (HTLs) at each frequency of each participant were adjusted by subtracting the age- and sex-specific HTL according to Table B.3 of ISO 1999 (ISO, 2013).

#### Definition of hearing loss

From the perspective of hearing protection, high-frequency noise-induced hearing loss (HFNIHL) was defined as adjusted HTL ≥ 30 dB, in either ear, at one or more of the HTLs 3 kHz, 4 kHz, and 6 kHz (Zhao et al., 2010; Chen et al., 2019; Zhou et al., 2020). Speech-frequency noise-induced hearing loss (SFNIHL) was defined as an average hearing threshold of HTL ≥ 26 dB in the better ear at speech frequencies of 0.5 kHz, 1 kHz, and 2 kHz (Zhou et al., 2020).

### Statistical analyses

Two study staff entered the data into an Excel spreadsheet for Windows Microsoft, WA, USA for analysis using the SPSS 19.0 program. Continuous variables were expressed as mean with standard deviation (mean ± SD). A one-way analysis of variance was used to compare continuous variables among the different types of work. The Chi-square test and Fisher’s exact test were used to compare the prevalence of HFNIHL (HFNIHL%) and the prevalence of SFNIHL (SFNIHL%) across different groups. We set the age of workers into six groups (≤ 25, 25–30, 30–35, 35–40, 40–45, and >45 years), and also set the noise exposure duration into six groups (≤3, 3–5, 5–10, 10–15, 15–20, and >20 years). The correlation between continuous variables was analyzed using the Pearson correlation method. Binary logistic regression analysis was used to analyze the odds ratio (OR) and 95% confidence interval values (CIs) of key factors affecting the HFNIHL% and SFNIHL% (as a categorical dependent variable). Differences with a *p* < 0.05 were considered statistically significant.

## Results

### Noise exposure and hearing loss associated with noise level and kurtosis

Table 1 shows the general information of noise exposure and hearing loss of manufacturing workers in this study. There were 1,050 participants in the present study; 751 (71.5%) of them were males. The mean age of the workers was 34.8 ± 9.8 years. The average noise exposure duration of participants was 7.3 ± 6.5 years.

**Table 1:** Noise exposure and hearing loss among different types of work in manufacturing industries (*n* = 1,050).

**Industry**	**Work type**	**n**	**Male (%)**	**Age (year)**	**ED (year)**	**L_Aeq.8h_[dB(A)]**		**Kurtosis**	**HFNIHL (%)**	**SFNIHL (%)**
						**Mean**	**≥85 (%)**
Textile		**321**	**165 (51.4)**	**34.0 ± 8.5**	**7.8 ± 6.0**	**93.6 ± 8.1**	**85.4**	**11.6 ± 13.3**	**206 (64.2)**	**18 (5.6)**
	Spinners	130	104 (80.0)	33.4 ± 8.4	9.4 ± 7.1	95.2 ± 9.2	84.6	10.4 ± 11.2	90 (69.2)	7 (5.4)
	Weavers	135	23 (17.0)	33.4 ± 8.6	6.6 ± 5.2	95.8 ± 3.3	99.3	8.1 ± 12.4	92 (68.1)	10 (7.4)
	Roller operator	56	38 (67.9)	36.9 ± 7.8	7.1 ± 3.4	84.5 ± 7.3	53.6	22.7 ± 14.0	24 (42.9)	1 (1.8)
Furniture		**318**	**303 (95.3)**	**33.6 ± 9.5**	**4.9 ± 4.9**	**88.9 ± 4.3**	**82.4**	**200.4 ± 161.7**	230 (72.3)	22 (6.9)
	Gun nailers	212	207 (97.6)	30.8 ± 8.1	4.3 ± 4.2	89.1 ± 4.4	84.4	246.4 ± 172.8	155 (73.1)	8 (3.8)
	Carpenters	106	96 (90.6)	39.2 ± 9.5	6.1 ± 5.9	88.5 ± 4.2	78.3	108.6 ± 78.5	75 (70.8)	14 (13.2)
General equipment		**411**	**283 (68.9)**	**36.3 ± 10.8**	**8.7 ± 7.5**	**86.4 ± 7.6**	**60.6**	**36.9 ± 52.5**	**241 (58.6)**	**38 (9.2)**
	Assemblers	152	61 (40.1)	34.6 ± 7.5	9.0 ± 7.0	86.9 ± 5.8	63.2	45.8 ± 77.8	96 (63.2)	10 (6.6)
	Metal processing workers	38	34 (89.5)	41.6 ± 11.0	15.4 ± 9.7	85.2 ± 6.7	42.1	34.2 ± 22.2	26 (68.4)	6 (15.8)
	Welders	34	33 (97.1)	41.0 ± 9.5	11.7 ± 8.4	89.8 ± 8.2	73.5	37.7 ± 30.4	24 (70.6)	3 (8.8)
	Polishers	27	23 (85.2)	48.3 ± 7.9	9.8 ± 4.8	91.4 ± 8.3	81.5	26.9 ± 32.3	21 (77.8)	3 (11.1)
	Forgers	42	40 (95.2)	45.5 ± 11.9	11.8 ± 6.8	85.6 ± 8.1	57.1	42.1 ± 33.2	27 (64.3)	6 (14.3)
	Stampers	64	50 (78.1)	27.0 ± 5.0	3.3 ± 3.4	86.1 ± 8.6	75.0	20.9 ± 19.3	26 (40.6)	7 (10.9)
	Carvers	54	42 (77.8)	32.1 ± 11.1	5.1 ± 5.3	82.2 ± 8.1	33.3	33.5 ± 27.7	21 (38.9)	3 (5.6)
Average		**1,050**	**751 (71.5)**	**34.8 ± 9.8**	**7.3 ± 6.5**	**89.4 ± 7.6**	**74.8**	**78.7 ± 124.9**	**677 (64.5)**	**78 (7.4)**

ED, Exposure duration; L_Aeq.8h_, The 8-h equivalent continuous A-weighted sound pressure level in decibels [dB(A)]; HFNIHL, High-frequency noise-induced hearing loss; SFNIHL, Speech-frequency noise-induced hearing loss.

#### Noise exposure among different types of work

The average L_Aeq.8h_ among the 1,050 workers was 89.4 ± 7.6 dB(A), ranging from 61.3 dB(A) to 105.6 dB(A). A total of 785 (74.8%) of workers from the manufacturing industry were occupationally exposed to noise levels above 85 dB(A), which exceeds the OEL in China. The proportion of workers exposed to occupational noise exceeding the OEL varied by industry and type of work (*p* < 0.05), as summarized in Table 1. For industries, 85.4% of workers from the textile industry were exposed to occupational noise above 85 dB(A), followed by the furniture manufacturing industry (82.4%) and the general equipment manufacturing industry (60.6%). The types of work with a higher L_Aeq.8h_ exceeding the OEL were weavers (99.3%), spinners (84.6%), gun nailers (84.4%), and polishers (81.5%; *p* < 0.05). There were statistically significant differences in kurtosis between different types of work (*p* < 0.001). The gun nailers were exposed to noise with the highest kurtosis (*β* = 246.4 ± 172.8), while the weavers were exposed to the lowest kurtosis (*β* = 8.1 ± 12.4), followed by the spinners (*β* = 10.4 ± 11.2; *p* < 0.05), as shown in Table 1. The correlation analysis across all the 1,050 subjects showed no correlation between L_Aeq.8h_ and kurtosis (*r* = −0.004, *p* = 0.885).

#### Prevalence of hearing loss among different types of work

The audiometric test results showed that the average HFNIHL% and SFNIHL% among workers exposed to manufacturing noise were 64.5% and 7.4%, respectively (Table 1). Significant differences were observed in average HFNIHL% and SFNIHL% among different types of work (for HFNIHL%, χ^2^ = 56.58, *p* < 0.001; for SFNIHL%, χ^2^ = 21.59, *p* = 0.028). The polishers had the highest HFNIHL%, followed by gun nailers, carpenters, and welders, while the metal processing workers had the highest SFNIHL%, followed by forgers and carpenters.

#### Principal characteristics of HFNIHL and SFNIHL prevalence

Results of the Chi-square test for the HFNIHL% and SFNIHL% in different groups were listed in Table 2. Sex, age group, noise exposure duration, L_Aeq.8h_, and kurtosis were all related to the HFNIHL%, while the SFNIHL% was only related to age and noise exposure duration. Male workers had a higher prevalence of HFNIHL than female workers (χ^2^ = 7.99, *p* = 0.005). Overall, the HFNIHL% increased with age (χ^2^ = 62.97, *p* < 0.001), although differences between some groups were not statistically significant = 62.97. The HFNIHL% of workers also increased with noise exposure duration (χ^2^ = 60.14, *p* < 0.001), as well as L_Aeq.8h_ level (χ^2^ = 47.05, *p* < 0.001). There were differences in the HFNIHL% of different kurtosis groups, and the HFNIHL% was the highest for those exposed to noise with kurtosis >50 (χ^2^ = 25.04, *p* < 0.001). The SFNIHL% increased with age and noise exposure duration (χ^2^ = 51.86, *p* < 0.001; χ^2^ = 13.47, *p* = 0.019, respectively).

**Table 2:** Principal characteristics of HFNIHL and SFNIHL among workers in manufacturing industries (*n* = 1,050).

**Factor**	**Group**	**n**	**HFNIHL**		**SFNIHL**	
			**n**	**%**	**n**	**%**
Sex	Male	751	504	67.1	59	7.9
	Female	299	173	57.9	19	6.4
			χ^2^ = 7.99, *p* = 0.005		χ^2^ = 0.70, *p* = 0.402	
Age (year)	≤25	201	91	45.3	10	5.0
	25–30	226	136	60.2	9	4.0
	30–35	180	114	63.3	7	3.9
	35–40	145	101	69.7	7	4.8
	40–45	146	114	78.1	13	8.9
	>45	152	121	79.6	32	21.1
			χ^2^ = 62.97, *p* < 0.001		χ^2^ = 51.86, *p* < 0.001	
ED (year)	≤3	398	206	51.8	22	5.5
	3–5	131	88	67.2	6	4.6
	5–10	278	186	66.9	23	8.3
	10–15	133	103	77.4	12	9.0
	15–20	68	58	85.3	7	10.3
	>20	42	36	85.7	8	19.1
			χ^2^ = 60.14, *p* < 0.001		χ^2^ = 13.47, *p* = 0.019	
L_Aeq.8h_ [dB(A)]	< 80	111	45	40.5	3	2.7
	80–85	154	85	55.2	9	5.8
	85–90	283	184	65.0	22	7.8
	≥90	502	363	72.3	44	8.8
			χ^2^ = 47.05, *p* < 0.001		χ^2^ = 5.52, *p* = 0.138	
Kurtosis	≤10	242	163	67.4	17	7.0
	10–50	445	250	56.2	30	6.7
	>50	363	264	72.7	31	8.5
			χ^2^ = 25.04, *p* < 0.001		χ^2^ = 1.01, *p* = 0.602	

HFNIHL, High-frequency noise-induced hearing loss; SFNIHL, Speech-frequency noise-induced hearing loss; ED, Exposure duration; L_Aeq.8h_, The 8-h equivalent continuous A-weighted sound pressure level in decibels [dB(A)].

### The effect of kurtosis on the association of noise exposure duration and noise intensity with hearing loss

#### The relationship between noise exposure duration and hearing loss at different kurtosis levels

**Figure 1:**
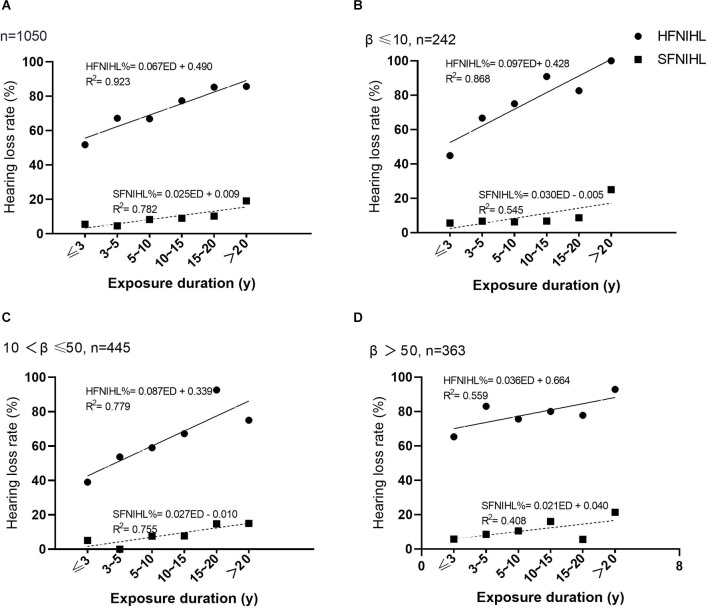
The relationship between noise exposure duration and hearing loss at different kurtosis levels. **(A)** The HFNIHL% and SFNIHL% of different noise exposure duration groups. **(B)** The HFNIHL% and SFNIHL% of different noise exposure duration groups when β ≤ 10. **(C)** The HFNIHL% and SFNIHL% of different noise exposure duration groups when β was between 10 and 50. **(D)** The HFNIHL% and SFNIHL% of different noise exposure duration groups when *β* > 50.

As shown in Figure 1, noise exposure duration promoted both HFNIHL and SFNIHL, but the effect of noise exposure duration on the HFNIHL% was more pronounced than that on the SFNIHL%. There was no significant difference in the SFNIHL prevalence among the noise exposure duration groups at different kurtosis levels after grouping according to the kurtosis level (for β ≤ 10, χ^2^ = 4.38, *p* = 0.496; for β greater than 10 and less than or equal to 50, χ^2^ = 9.72, *p* = 0.084; and for β > 50, χ^2^ = 7.06, *p* = 0.216). There were statistical differences of the HFNIHL% between noise exposure duration groups at different kurtosis levels (for β ≤ 10, χ^2^ = 39.03, *p* < 0.001; for β greater than 10 and less than or equal to 50, χ^2^ = 37.54, *p* < 0.001; and for β > 50, χ^2^ = 11.39, *p* < 0.001). The HFNIHL% of workers exposed to noise for 3 years or less was 44.9%, 39.0%, and 64.1% when β ≤ 10, 10–50, and >50, respectively.

There were significant differences in HFNIHL% between kurtosis levels when the noise exposure duration was less than 10 years. The HFNIHL% with β > 50 was always significantly higher than that of those with β ≤ 50 for workers with ED ≤10 years (for ED ≤ 3 years, χ^2^ = 22.53, *p* < 0.001; for ED between 3 and 5 years, χ^2^ = 8.30, *p* = 0.004; for ED between 5 and 10 years, χ^2^ = 4.23, *p* = 0.040). In contrast, the HFNIHL% at different kurtosis levels were not statistically different when the noise exposure duration exceeded 10 years (*p* > 0.05). The SFNIHL% among 1,050 workers also did not differ by kurtosis level regardless of the duration of noise exposure (*p* > 0.05).

#### The association of noise intensity and kurtosis with hearing loss at different kurtosis levels

**Figure 2:**
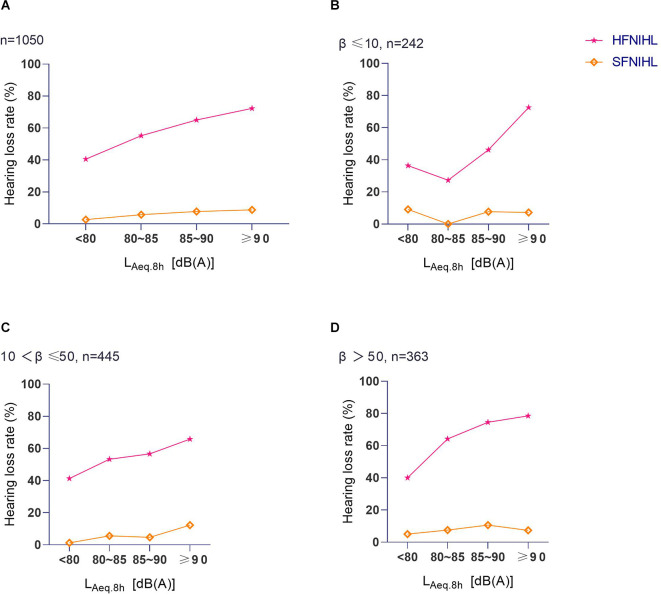
The relationship between L_Aeq.8h_ and hearing loss at different kurtosis levels. **(A)** The HFNIHL% and SFNIHL% of different L_Aeq.8h_ groups. **(B)** The HFNIHL% and SFNIHL% of different L_Aeq.8h_ groups when β ≤ 10. **(C)** The HFNIHL% and SFNIHL% of different L_Aeq.8h_groups when β was between 10 and 50. **(D)** The HFNIHL% and SFNIHL% of different L_Aeq.8h_ groups when β > 50.

Overall, the HFNIHL% increased with L_Aeq.8h_ levels, while the SFNIHL% showed no difference between different L_Aeq.8h_ levels (χ^2^ = 5.52, *p* = 0.138). The influence of noise intensity on the HFNIHL% and SFNIHL% at different kurtosis levels was additionally analyzed; results were summarized in Figure 2. At each kurtosis level, there were statistical differences in the HFNIHL% among different L_Aeq.8h_ groups (for β ≤ 10, χ^2^ = 17.96, *p* < 0.001; for β greater than 10 and less than or equal to 50, χ^2^ = 12.98, *p* = 0.005; and for β >50, χ^2^ = 15.50, *p* = 0.001). The HFNIHL% of workers at the same noise level increased with the kurtosis level when the L_Aeq.8h_ was between 80 dB(A) and 90 dB(A) (*p* < 0.05). However, there were no statistical differences in the HFNIHL% at different kurtosis levels when workers were exposed to noise with L_Aeq.8h_ < 80 dB(A) and L_Aeq.8h_ ≥90 dB(A) (for L_Aeq.8h_ < 80 dB(A), χ^2^ = 0.10, *p* = 0.950; for L_Aeq.8h_ ≥ 90 dB(A), χ^2^ = 6.01, *p* = 0.050). At the same time, there were still no statistical differences in the SFNIHL% between L_Aeq.8h_ levels after stratifying by the kurtosis level (*p* > 0.05).

### NIPTS associated with noise exposure characteristics

#### Symmetrical and notching shape of NIPTS curves among different types of work

**Figure 3:**
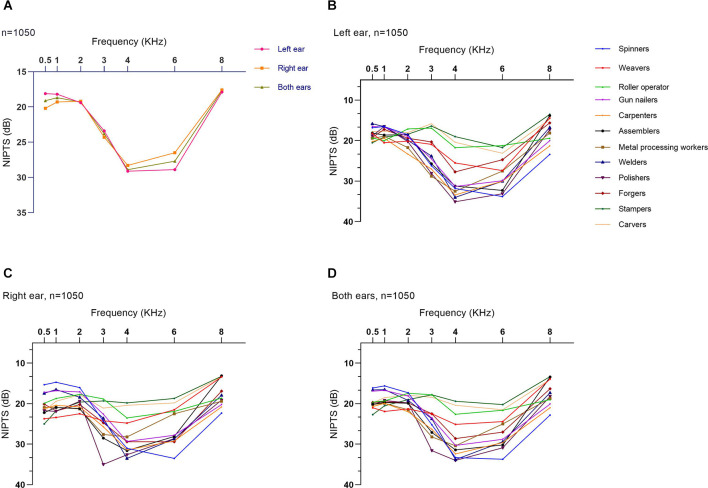
Symmetrical and notching shape of average NIPTS curves for manufacturing workers. **(A)** Average NIPTS curves of the left ear, right ear, and both ears of manufacturing workers. **(B)** Average NIPTS curves of the left ears of different types of work. **(C)** Average NIPTS curves of the right ears of different types of work. **(D)** Average NIPTS curves of both ears of different types of work.

The mean NIPTS of the speech frequencies (19.1 ± 7.0 dB HL) was lower than that of the high frequencies (24.1 ± 13.3 dB HL; *p* < 0.05). Figure 3A shows the curves of the average NIPTS of manufacturing workers. The shapes of the average NIPTS curves of left and right ears almost overlapped across the speech and high frequencies, with a classic “V” shape notch. The average NIPTS increased with the test frequencies at 0.5 kHz to 4 kHz. After exhibiting the highest level of average NIPTS at 4 kHz, it then gradually decreased with the test frequencies from 4 kHz to 8 kHz. The mean NIPTS of the speech frequencies between different types of work was statistically different (*p* < 0.001), and that of the high frequencies. The NIPTS curves of different types of work were different, as shown in Figures 3B,C,D. The NIPTS curves of stampers, carvers, roller operators, and weavers had a shallow depth of “V”, while the curves of polishers, welders, gun nailers, assemblers, and metal processing workers were a deeper “V” shape.

#### Association of average NIPTS for manufacturing workers with noise exposure duration, noise intensity, and kurtosis levels

**Figure 4:**
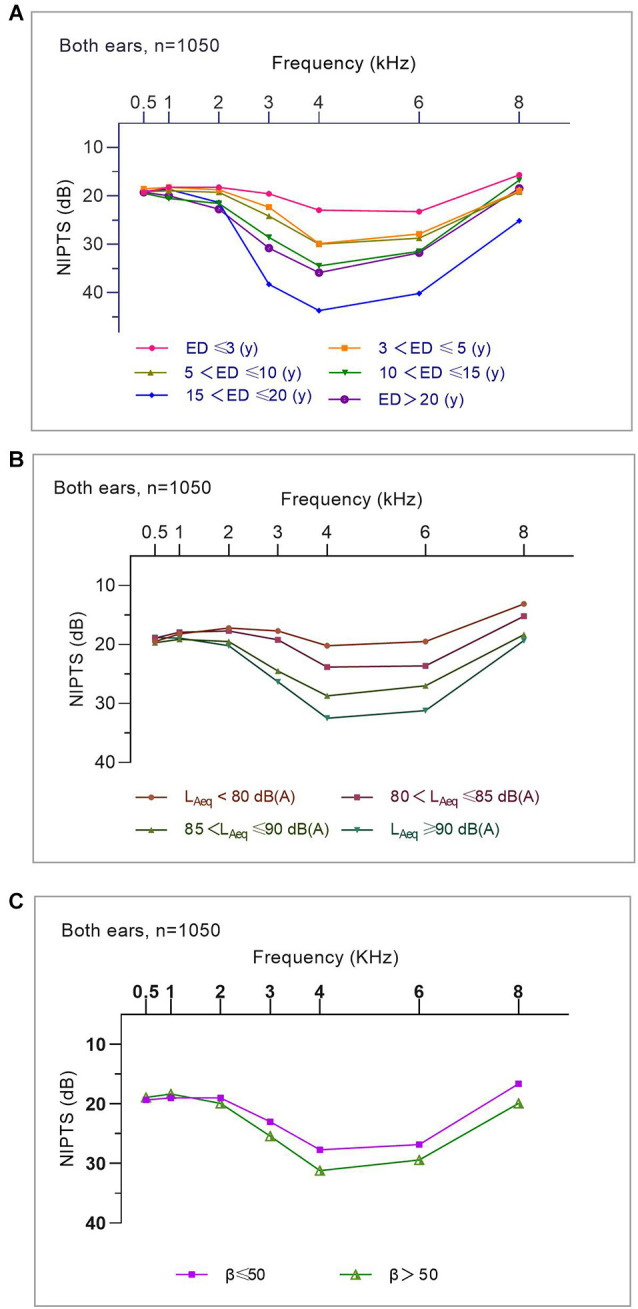
The association of average NIPTS curves for manufacturing workers with noise exposure duration, noise intensity, and kurtosis levels. **(A)** Average NIPTS curves of workers with different noise exposure duration. **(B)** Average NIPTS curves of workers exposed to noise at different L_Aeq.8h_ levels. **(C)** Average NIPTS curves of workers exposed to noise at different kurtosis levels.

Figure 4 displays the “V”-shaped average NIPTS curves of manufacturing workers at different levels of noise exposure duration, L_Aeq.8h_, and kurtosis. The average NIPTS of speech frequencies did not show a significant trend of increasing with noise exposure duration and L_Aeq.8h_ (*p* > 0.05). The depth of the “V” shape notch at 4 kHz in the NIPTS curves deepened with the noise exposure duration when the exposure duration was within 15 years but did not gradually deepen with the exposure duration when the ED > 15 years (Figure 4A). The notch depth of the average NIPTS curves deepened gradually with L_Aeq.8h_ levels, especially for frequencies of 3 kHz to 6 kHz (*p* < 0.001; Figure 4B). Figure 4C illustrated that the high-frequency “V”-shaped hearing valley of curves of workers with β > 50 was significantly deeper than that of workers exposed to noise with β ≤ 50 (*p* < 0.05). However, there was no difference in the shift of speech frequency hearing threshold at different kurtosis levels (*p* > 0.05).

#### The influence of kurtosis on the association of average NIPTS for manufacturing workers with noise exposure duration and noise intensity

**Figure 5:**
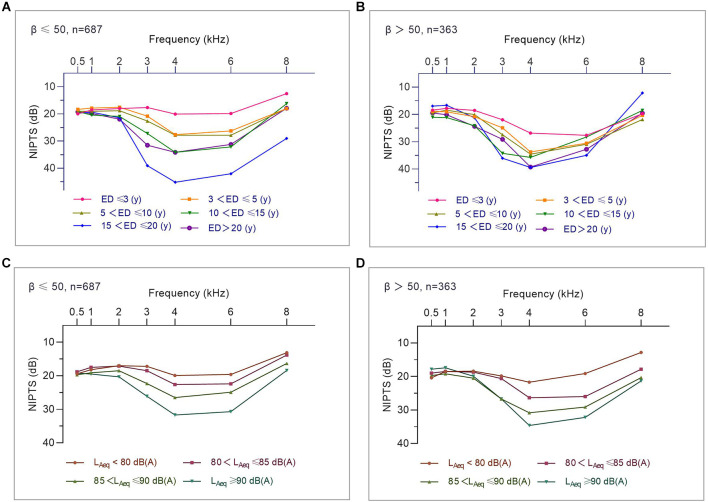
The influence of kurtosis on the association of average NIPTS curves for manufacturing workers with noise exposure duration and noise intensity. **(A)** Average NIPTS curves of workers with different noise exposure duration when β ≤ 50. **(B)** Average NIPTS curves of workers with different noise exposure duration when β > 50. **(C)** Average NIPTS curves of workers exposed to noise at different L_Aeq.8h_ levels when β ≤ 50. **(D)** Average NIPTS curves of workers exposed to noise at different L_Aeq.8h_levels when *β* > 50.

Figures 5A,B shows that the V-shaped dips of the curve (i.e., hearing threshold shift at 3 kHz, 4 kHz, and 6 kHz) were generally deeper when *β* > 50 than those exposed to noise with β ≤ 50 for workers with ED ≤ 10 years. However, this effect was not shown among workers with ED > 10 years. This result suggested that the effect of kurtosis on the association of noise exposure duration and mean NIPTS was more pronounced for workers with ED ≤ 10 years than those with ED > 10 years. Furthermore, the notch of NIPTS curves at high frequencies for workers exposed to noise with L_Aeq.8h_ level between 80 dB(A) to 90 dB(A) was significantly deeper than that of workers exposed to noise with β ≤ 50, as shown in Figures 5C,D. This effect was not shown among workers exposed to noise with L_Aeq.8h_ < 80 dB(A) or L_Aeq.8h_ ≥ 90 dB(A). It indicates that the effect of kurtosis on the association of noise intensity and the average NIPTS in workers exposed to noise with L_Aeq.8h_ between 80 dB(A) to 90 dB(A) was more significant than in those exposed to noise with L_Aeq.8h_ < 80 dB(A) or L_Aeq.8h_ ≥ 90 dB(A).

### Binary logistic regression analysis of the association between key factors and the HFNIHL and SFNIHL prevalence

As demonstrated in Table 3, after controlling for the influence of other factors, the HFNIHL% of workers exposed to manufacturing noise was related to sex, age, noise exposure duration, kurtosis, and noise intensity (L_Aeq.8h_). Male workers had a 55.7% higher risk of HFNIHL than female workers (OR = 1.557, 95%CI = 1.141–2.124). The risk of HFNIHL increased with age (OR = 1.033, 95%CI = 1.014–1.052) and noise exposure duration (OR = 1.072, 95%CI = 1.038–1.107). Both kurtosis and noise intensity contributed to an increase risk of HFNIHL (for kurtosis, OR = 1.002, 95%CI = 1.001–1.003; for L_Aeq.8h_, OR = 1.064, 95% CI = 1.044–1.084).

**Table 3:** Binary logistic regression analysis showing association between key factors and the prevalence of HFNIHL and SFNIHL among workers in manufacturing industries (*n* = 1,050).

**Factor**	**HFNIHL**					**SFNIHL**				
	**B**	**SE**	**Wald χ^2^**	***p***	**OR (95% CI)**	**B**	**SE**	**Wald χ^2^**	***p***	**OR (95% CI)**
Male	0.443	0.158	7.808	0.005	1.557 (1.141–2.124)	0.123	0.292	0.179	0.673	1.131 (0.639–2.003)
Age (year)	0.032	0.009	12.39	0.004	1.033 (1.0147–1.052)	0.069	0.014	25.750	<0.001	1.071 (1.043–1.100)
ED (year)	0.069	0.016	17.934	<0.001	1.072 (1.038–1.107)	0.004	0.018	0.053	0.818	1.004 (0.969–1.041)
Kurtosis	0.002	0.006	10.088	0.002	1.002 (1.001–1.003)	0.001	0.001	0.911	0.340	1.001 (0.999–1.003)
L_Aeq.8h_ [dB(A)]	0.062	0.009	42.28	<0.001	1.064 (1.044–1.084)	0.026	0.018	2.145	0.143	1.026 (0.991–1.062)

HFNIHL, High-frequency noise-induced hearing loss; SFNIHL, Speech-frequency noise-induced hearing loss; SE: Standard error; OR, Odds ratio; CI, Confidence interval; ED, Exposure duration; L_Aeq.8h_, The 8-h equivalent continuous A-weighted sound pressure level in decibels [dB(A)].

Unlike the HFNIHL, after controlling other factors, the SFNIHL risk of workers exposed to manufacturing noise was only related to age (OR = 1.071, 95%CI = 1.043–1.100). There were no statistical associations between the SFNIHL and sex, noise exposure duration, kurtosis, and noise intensity (L_Aeq.8h_; *p* > 0.05).

## Discussion

As a type of progressive sensorineural hearing loss, NIHL has been a global public health problem for a long time (Basner et al., 2014). Hearing loss caused by occupational noise exposure in the workplace is a worldwide health problem. In China, 67.56% of the diagnosed cases of occupational otolaryngological and stomatological diseases were from the manufacturing industry in 2020 (Zheng et al., 2021). The equal energy hypothesis, which has been the basis of the noise evaluation metric (*L*_Aeq_; ISO, 2013), implies that hearing loss is independent of the temporal characteristics of noise. Nonetheless, many industrial noise environments are non-Gaussian noise (Zhou et al., 2020). Studies have revealed that complex noise exposure could cause a greater risk of NIHL than Gaussian noise (Zhao et al., 2010; Goley et al., 2011; Suter, 2017; Zhang et al., 2021b). The energy metric of noise alone does not apply to the assessment of hearing loss caused by non-Gaussian noise in the workplace and the temporal metric of noise should be considered to be a supplemental indicator of NIHL assessment (Davis et al., 2009, 2012; Seixas et al., 2012; Xie et al., 2016; Zhang et al., 2022).

This study investigated the epidemiological characteristics of hearing loss due to kurtosis-based noise exposure in manufacturing workers. Table 1 showed that manufacturing workers occupationally exposed to noise with an average L_Aeq.8h_ of 89.4 ± 7.6 dB(A), 74.8% of them were exposed to noise exceeding the OEL of 85 dB(A). Our findings were consistent with those of other studies. In South Korea, more than 90% of workplace noise levels exceeded 85 dB(A) (Kim, 2010). Zhang et al. (2021b) investigated noise exposure levels of workers in six Chinese manufacturing industries, in which 77.6% of the workers were exposed to noise levels higher than 85 dB(A). The noise intensity metric (L_Aeq.8h_) and the noise temporal metric (kurtosis) were distributed differently in different types of work in this study. The weavers, spinners, and gun nailers were exposed to higher L_Aeq.8h_ levels than other types of work, while gun nailers and weavers were exposed to the highest and lowest noise kurtosis levels, respectively. These results indicated that the noise intensity and kurtosis were independent parameters, which was supported by the result of the correlation analysis. Similar results have been found in previous studies (Chen et al., 2019; Zhou et al., 2021).

The prevalence of hearing loss among workers remains high due to the high level of noise exposure. In the United States, approximately 15% of workers have experienced NIHL (Shargorodsky et al., 2010). A meta-analysis study found that the occupational NIHL prevalence in China was 21.3%, of which 30.2% and 9.0% accounted for the prevalence of HFNIHL and SFNIHL, respectively (Zhou et al., 2020). Likewise, the audiometric test results of this study indicated that the average HFNIHL% (64.5%) was much higher than the average SFNIHL% (7.4%) for workers occupationally exposed to manufacturing noise. Significant differences were observed in the average HFNIHL% and SFNIHL% among different types of work. Meanwhile, although both the mean NIPTS for speech frequencies (19.1 ± 7.0 dBHL) and high frequencies (24.1 ± 13.3 dB HL) were within the normal limits, workers of different types of work had their own unique NIPTS curves, which has been revealed in previous studies (Chen et al., 2019; Zhang et al., 2021b).

The average age of participants in this study was 34.8 ± 9.8 years. Age-related hearing loss, defined as a progressive, bilateral, symmetrical age-related sensorineural hearing loss, is a complex disorder that results from the cumulative effects of aging on the auditory system (Bowl and Dawson, 2019). The effect of age on hearing loss is most pronounced at the higher frequencies and a lifetime of noise overexposure also significantly worsens age-related hearing loss (Wu et al., 2020). In this study, the risk of HFNIHL and SFNIHL both increased with age. In addition, male workers experienced a higher risk of HFNIHL than female workers. Some studies reported similar results that age and sex were risk factors for NIHL, even though the hearing thresholds were already adjusted by age and sex based on Annex B Table B.3 in the ISO 1999 (ISO, 2013; Lie et al., 2014; Bolm-Audorff et al., 2020; Zhang et al., 2021b).

The prevalence of NIHL increased with exposure duration, especially during the first 10 years of noise exposure (Bauer et al., 1991; Zhou et al., 2020). The average noise exposure duration of manufacturing workers recruited in this study was 7.3 ± 6.5 years. After controlling other risk factors, the odds of the HFNIHL increased 7.2% with noise exposure duration as shown in Table 3. A cross-sectional study in eastern Saudi Arabia revealed that noise exposure is the primary cause of hearing loss (Ahmed et al., 2001). The prevalence of HFNIHL was associated with both noise intensity and its temporary structure as detected in the binary logistic regression results, which were supported by previous studies. Chen et al. (2019) studied the prevalence and determinants of NIHL among workers in the automotive industry and found the prevalence of NIHL increased with the increasing noise energy levels including L_Aeq.8h_. Zhang et al. (2021b) also found the L_Aeq.8h_ has the highest contribution to NIHL.

In recent years, researchers realized that in addition to the noise intensity, the temporal metric plays an important role in leading NIHL. A meta-analysis study in China found the overall weighted OR for complex noise was 1.95, which demonstrated that exposure to complex noise could lead to greater hearing loss than exposure to Gaussian noise (Zhou et al., 2020). Other epidemiological studies have also suggested that the kurtosis metric should be considered when evaluating noise exposure and the risk and cause of NIHL (Qiu et al., 2006; Xie et al., 2016; Shi et al., 2021; Zhang et al., 2021a, b). Our findings indicated that kurtosis was an independent risk factor for the HFNIHL% and it could make the NPTS curve of manufacturing workers a deeper V-shape.

The results of this study further uncovered the effect of kurtosis on the association of exposure duration and noise intensity with NIHL under certain conditions. Kurtosis was able to deepen the hearing notch at high frequencies and accelerate the formation of early hearing loss. The HFNIHL% increased with kurtosis level (β > 50 vs. β ≤ 50) when the noise exposure duration was within 10 years. Conversely, the kurtosis did not affect the relationship between the HFNIHL% and noise exposure duration when ED >10 years. A similar result was obtained from another study, which suggested that ISO 1999 underestimated the noise exposure duration of NIPTS by less than or equal to 10 years (Zhang et al., 2021c). The present study also identified that the HFNIHL% of workers exposed to noise at the same intensity level increased with the kurtosis level when the L_Aeq.8h_ was between 80 and 90 dB(A). However, the effect of L_Aeq.8h_ on HFNIHL was not affected by kurtosis when workers were occupationally exposed to noise with L_Aeq.8h_ < 80 dB(A) or ≥ 90 dB(A). Even if the L_Aeq.8h_ levels meet the OEL, the HFNIHL risk for workers exposed to high kurtosis noise may still be unacceptable, especially those exposed to noise with L_Aeq.8h_at 80–85 dB(A). These results suggested that the OEL of 85 dB(A) regardless of the kurtosis of noise should be reconsidered. It was consistent with the results of previous studies. Zhang et al. suggested the uncertainty of the OEL of 85 dB(A) might be related to noise exposure with a complex temporal structure, for the NIHL% of workers exposed to noise with L_Aeq.8h_ level of 80–85 dB(A) with a high kurtosis (β > 100) was significantly higher than those exposed to noise at the same level of L_Aeq.8h_ with *β* < 100 (Zhang et al., 2021b).

This study had several limitations. The number of participants in some types of work recruited in this study may result in limited numbers of certain categories after grouping by variables, which may affect the statistical efficiency of some analyses. Therefore, we grouped kurtosis less than some similar studies to reduce the impact of this limitation, and the results can still basically draw its influence on hearing loss and its risk factors. Additionally, the majority of participants of this study were young men, whose exposure duration might be shorter than elder workers. As a result, the representativeness of the sample in the manufacturing industry might be insufficient. Another limitation of this study was that it included only a limited number of industries and types of work, which may be slightly under-represented in the broad range of noise types in different manufacturing industries. More participants from various industries including more types of work should be recruited in future studies to improve representation.

## Conclusion

The results of this study indicated that: (1) the HFNIHL among manufacturing workers is associated with sex, age, noise exposure duration, L_Aeq.8h_,and kurtosis, while the SFNIHL is associated with age; (2) the kurtosis strengthens the association of noise exposure duration and noise intensity with hearing loss among workers exposed to noise with L_Aeq.8h_ between 80 and 90 dB(A) or with ED less than10 years; (3) an acoustic energy metric is necessary but not sufficient to evaluate the risk of NIHL; (4) the temporal structure of noise such as kurtosis is an additional metric should be considered when evaluating the risk of NIHL by complex noise. These findings would be better replicated using data from a larger sample of workers exposed to a wide range of noise types to provide more information on NIHL in future studies.

## Data Availability Statement

The raw data supporting the conclusions of this article will be made available by the authors, without undue reservation.

## Ethics Statement

The studies involving human participants were reviewed and approved by The Medical Ethics Committee of Zhejiang Center for Disease Control and Prevention. The patients/participants provided their written informed consent to participate in this study.

## Author Contributions

LZ, XR, and TW: investigation, formal analysis and writing—original draft. HX: methodology and investigation. YH: investigation and data curation. ZS, JX, and JZ: formal analysis and visualization. PX, FW, and YZ: investigation, data curation, and formal analysis. MZ: conceptualization, funding acquisition, writing—review and editing. HZ: funding acquisition, writing—review and editing, and supervision. All authors contributed to the article and approved the submitted version.

## Funding

This research was funded by the Zhejiang Provincial Key Research and Development Project (grant number: 2015C03039); the Zhejiang Provincial Program for the Cultivation of High-Level Innovative Health Talents, Zhejiang Province, China; the Pre-research Project on Occupational Health Standards (20210102); and the Health Commission of Zhejiang Province (2019KY057).
